# Model-independent searches of new physics in DARWIN with deep learning

**DOI:** 10.1140/epjc/s10052-025-15161-2

**Published:** 2026-03-26

**Authors:** XLZD Collaboration, J. Aalbers, K. Abe, M. Adrover, S. Ahmed Maouloud, L. Althueser, D. W. P. Amaral, B. Andrieu, E. Angelino, D. Antón Martin, B. Antunovic, E. Aprile, M. Babicz, D. Bajpai, M. Balzer, E. Barberio, L. Baudis, M. Bazyk, N. F. Bell, L. Bellagamba, R. Biondi, Y. Biondi, A. Bismark, C. Boehm, K. Boese, R. Braun, A. Breskin, S. Brommer, A. Brown, G. Bruni, R. Budnik, C. Cai, C. Capelli, A. Chauvin, A. P. Cimental Chavez, A. P. Colijn, J. Conrad, J. J. Cuenca-García, V. D’Andrea, L. C. Daniel Garcia, M. P. Decowski, A. Deisting, C. Di Donato, P. Di Gangi, S. Diglio, M. Doerenkamp, G. Drexlin, K. Eitel, A. Elykov, R. Engel, A. D. Ferella, C. Ferrari, H. Fischer, T. Flehmke, M. Flierman, K. Fujikawa, W. Fulgione, C. Fuselli, P. Gaemers, R. Gaior, M. Galloway, F. Gao, N. Garroum, R. Giacomobono, F. Girard, R. Glade-Beucke, F. Glück, L. Grandi, J. Grigat, R. Größle, H. Guan, M. Guida, P. Gyorgy, R. Hammann, V. Hannen, S. Hansmann-Menzemer, N. Hargittai, A. Higuera, C. Hils, K. Hiraoka, L. Hoetzsch, N. F. Hood, M. Iacovacci, Y. Itow, J. Jakob, R. S. James, F. Joerg, F. Kahlert, Y. Kaminaga, M. Kara, P. Kavrigin, S. Kazama, M. Keller, P. Kharbanda, B. Kilminster, M. Kleifges, M. Klute, M. Kobayashi, D. Koke, A. Kopec, B. von Krosigk, F. Kuger, L. LaCascio, H. Landsman, R. F. Lang, L. Levinson, I. Li, A. Li, S. Li, S. Liang, Z. Liang, Y. -T. Lin, S. Lindemann, M. Lindner, K. Liu, J. Loizeau, F. Lombardi, J. Long, J. A. M. Lopes, G. M. Lucchetti, T. Luce, Y. Ma, C. Macolino, J. Mahlstedt, B. Maier, A. Mancuso, L. Manenti, F. Marignetti, K. Martens, J. Masbou, E. Masson, S. Mastroianni, A. Melchiorre, J. Menéndez, M. Messina, B. Milosovic, S. Milutinovic, K. Miuchi, R. Miyata, A. Molinario, C. M. B. Monteiro, K. Morå, S. Moriyama, E. Morteau, Y. Mosbacher, J. Müller, M. Murra, J. L. Newstead, K. Ni, C. O’Hare, U. Oberlack, M. Obradovic, I. Ostrowskiy, S. Ouahada, B. Paetsch, Y. Pan, M. Pandurovic, Q. Pellegrini, R. Peres, F. Piastra, J. Pienaar, M. Pierre, G. Plante, T. R. Pollmann, L. Principe, J. Qi, K. Qiao, J. Qin, M. Rajado, D. Ramírez García, A. Ravindran, A. Razeto, L. Sanchez, P. Sanchez-Lucas, G. Sartorelli, A. Scaffidi, J. Schreiner, P. Schulte, H. Schulze Eißing, M. Schumann, A. Schwenck, L. Scotto Lavina, M. Selvi, F. Semeria, P. Shagin, S. Sharma, W. Shen, S. Y. Shi, T. Shimada, H. Simgen, R. Singh, M. Solmaz, O. Stanley, M. Steidl, A. Stevens, A. Takeda, P.-L. Tan, D. Thers, T. Thümmler, F. Tönnies, F. Toschi, G. Trinchero, R. Trotta, C. D. Tunnell, P. Urquijo, M. Utoyama, K. Valerius, S. Vecchi, S. Vetter, G. Volta, D. Vorkapic, W. Wang, K. M. Weerman, C. Weinheimer, M. Weiss, D. Wenz, M. Wilson, C. Wittweg, J. Wolf, V. H. S. Wu, S. Wüstling, M. Wurm, Y. Xing, D. Xu, Z. Xu, M. Yamashita, L. Yang, J. Ye, L. Yuan, G. Zavattini, M. Zhong, K. Zuber

**Affiliations:** 1 Nikhef and the University of Groningen, Van Swinderen Institute, Groningen, 9747AG The Netherlands; 2 Kamioka Observatory, Institute for Cosmic Ray Research, and Kavli Institute for the Physics and Mathematics of the Universe (WPI), University of Tokyo,Hida, Higashi-Mozumi, Kamioka, Gifu, 506-1205 Japan; 3 Physik-Institut, University of Zürich, Zürich, 8057 Switzerland; 4 LPNHE, Sorbonne Université,CNRS/IN2P3, Paris, 75005 France; 5 Institute for Nuclear Physics, University of Münster, Münster, 48149 Germany; 6 Department of Physics and Astronomy, Rice University, Houston, TX 77005 USA; 7 INAF-Astrophysical Observatory of Torino, Department of Physics, University of Torino and INFN-Torino, Tourin, 10125 Italy; 8 INFN-Laboratori Nazionali del Gran Sasso and Gran Sasso Science Institute, L’Aquila, 67100 Italy; 9 Department of Physics, Enrico Fermi Institute & Kavli Institute for Cosmological Physics, University of Chicago, Chicago, IL 60637 USA; 10 Vinca Institute of Nuclear Science, University of Belgrade, Mihajla Petrovica Alasa 12-14, Belgrade, Serbia; 11 Physics Department, Columbia University, New York, NY 10027 USA; 12 Department of Physics & Astronomy, University of Alabama, Tuscaloosa, AL 34587-0324 USA; 13 Institute for Data Processing and Electronics, Karlsruhe Institute of Technology, Karlsruhe, 76021 Germany; 14 ARC Centre of Excellence for Dark Matter Particle Physics, School of Physics, The University of Melbourne, Vic, 3010 Australia; 15 SUBATECH, IMT Atlantique, CNRS/IN2P3, Nantes Université, Nantes, 44307 France; 16 Department of Physics and Astronomy, University of Bologna and INFN-Bologna, Bologna, 40126 Italy; 17 Max-Planck-Institut für Kernphysik, Heidelberg, 69117 Germany; 18 Institute for Astroparticle Physics, Karlsruhe Institute of Technology, Karlsruhe, 76021 Germany; 19 School of Physics, The University of Sydney, Camperdown, Sydney, NSW 2006 Australia; 20 Department of Particle Physics and Astrophysics, Weizmann Institute of Science, Rehovot, 7610001 Israel; 21 Institute of Experimental Particle Physics, Karlsruhe Institute of Technology, Karlsruhe, 76021 Germany; 22 Physikalisches Institut, Universität Freiburg, Freiburg, 79104 Germany; 23 Physikalisches Institut, Universität Freiburg, Freiburg, (Now at Sheffield), 79104 Germany; 24 Department of Physics and Astronomy, University of Sheffield, Sheffield, S3 7RH UK; 25 Department of Physics & Center for High Energy Physics, Tsinghua University, Beijing, 100084 People’s Republic of China; 26 Physikalisches Institut, Universität Heidelberg, Heidelberg, Germany; 27 Nikhef and the University of Amsterdam, Science Park, Amsterdam, 1098XG The Netherlands; 28 Oskar Klein Centre, Department of Physics, Stockholm University, AlbaNova, Stockholm, 10691 Sweden; 29 Institut für Physik & Exzellenzcluster PRISMA+, Johannes Gutenberg-Universität Mainz, Mainz, 55099 Germany; 30 Department of Physics and Chemistry, University of L’Aquila, L’Aquila, 67100 Italy; 31 Kobayashi-Maskawa Institute for the Origin of Particles and the Universe, and Institute for Space-Earth Environmental Research, Nagoya University, Furo-cho, Chikusa-ku, Nagoya, Aichi, 464-8602 Japan; 32 Department of Physics “Ettore Pancini”, University of Napoli and INFN-Napoli, Napoli, 80126 Italy; 33 Department of Physics and Astronomy, Purdue University, West Lafayette, IN 47907 USA; 34 Albert Einstein Center for Fundamental Physics, Institute for Theoretical Physics, University of Bern, Sidlerstrasse 5, Bern, 3012 Switzerland; 35 Department of Physics, University of California San Diego, La Jolla, CA 92093 USA; 36 Department of Physics and Astronomy, University College London (UCL), London, WC1E 6BT UK; 37 Department of Physics & Astronomy, Bucknell University, Lewisburg, PA USA; 38 Kirchhoff-Institut für Physik, Universität Heidelberg, Heidelberg, Germany; 39 Department of Physics, School of Science, Westlake University, Hangzhou, 310030 People’s Republic of China; 40 School of Science and Engineering, The Chinese University of Hong Kong, Shenzhen, Guangdong, 518172 People’s Republic of China; 41 LIBPhys, Department of Physics, University of Coimbra, Coimbra, 3004-516 Portugal; 42 Physics Department, Imperial College London Blackett Laboratory, London, SW7 2AZ UK; 43 Department of Quantum Physics and Astrophysics and Institute of Cosmos Sciences, University of Barcelona, Barcelona, 08028 Spain; 44 Department of Physics, Kobe University, Kobe, Hyogo, 657-8501 Japan; 45 Theoretical and Scientific Data Science, Scuola Internazionale Superiore di Studi Avanzati (SISSA), Trieste, 34136 Italy; 46 Department of Physics, Technische Universitaät Darmstadt, Darmstadt, 64289 Germany; 47 INFN-Ferrara and Dip. di Fisica e Scienze della Terra, Università di Ferrara, Ferrara, 44122 Italy; 48 Technische Universität Dresden, Dresden, 01069 Germany; 49 University of Banja Luka, Banja Luka, 78000 Bosnia and Herzegovina; 50 INFN-Roma Tre, Roma, 00146 Italy; 51 Coimbra Polytechnic - ISEC, Coimbra, 3030-199 Portugal; 52 University of Grenada, Grenada, Spain; 53 Department of Physics and Astronomy, University of Sheffield, Sheffield, S3 7RH UK

## Abstract

We present a deep learning pipeline to perform a model-independent, likelihood-free search for anomalous (i.e., non-background) events in the proposed next-generation multi-ton scale liquid xenon-based direct detection experiment, DARWIN. We train an anomaly detector comprising a variational autoencoder (VAE) and a classifier on high-dimensional simulated detector response data and construct a 1D anomaly score to reject the background-only hypothesis in the presence of an excess of non-background-like events. We use simulated validation data to determine the power of the method to reject the background-only hypothesis in the presence of a WIMP dark matter signal, without any model-dependent assumption about the nature of the signal. We show that our neural networks learn relevant features of the events from low-level, high-dimensional detector outputs, avoiding lossy and computationally expensive compression into lower-dimensional observables. Our approach is complementary to the usual likelihood-based analysis, in that it reduces the reliance on many of the corrections and cuts that are traditionally part of the analysis chain, with the potential of achieving higher accuracy and significant reduction of analysis time. We envisage the methodology presented in this work augmenting or complementing likelihood-based and other data-driven methods currently utilized in the DARWIN (and in the future, XLZD) analysis pipeline.

## Introduction

A promising method for investigations of the ever-elusive dark matter sector involves seeking excess nuclear recoils in subterranean detectors, a strategy known as direct detection (DD) [1]. Over the years, a number of xenon (XENONnT [2], LUX-ZEPLIN (LZ) [3], PandaX[4]) and argon (DEAP-3600 [5], DarkSide-20k [6], ArDM [7]) ton-scale experiments have steadily enhanced the sensitivity to physics beyond the standard model (BSM), and this effort is expected to continue, with plans for a next-generation dark matter and neutrino observatory. While earlier designs for a ‘dark matter WIMP search with liquid xenon’ observatory (DARWIN) [8, 9] aimed at an active liquid xenon target mass of 40 tons, the recently formed XLZD Collaboration proposes an even more ambitious target mass of 60–80 tons [10]. While the design of the XLZD experiment is being developed, this paper focuses on DARWIN, a well-defined proposal for a large-scale observatory using a xenon dual-phase time projection chamber (TPC) to study phenomena requiring low-background conditions. DARWIN aims to be sensitive to weakly interacting massive particle (WIMP) dark matter as well as neutrinoless double beta decay, axion-like particles, and any other BSM particles that would manifest through significant interaction with a xenon target. The aim of this work is to introduce a signal model-agnostic, deep learning-based analysis pipeline, offering a complementary and alternative approach to the standard likelihood-based analysis chain in such a detector. The benefits of this approach are that it enables a fuller exploitation of the detector readout data, without the information loss potentially incurred in using only hand-crafted summary statistics (such as cS1 and cS2, the corrected prompt primary scintillation and secondary electroluminescence of ionized electrons signals, respectively), and that it can include in the pipeline any physics effect that can be faithfully simulated, including systematics.

Machine learning (ML) has emerged as a powerful tool within the physics community, and its relevance to DM phenomenology has been growing rapidly [11–15].

Unsupervised machine learning has been increasingly employed in collider physics to identify anomalies in data, as demonstrated in several recent studies [16–27], with early example applications on simulated events of CMS and ATLAS already in Refs. [28, 29], as well as Ref. [30], where an “anomaly awareness” algorithm is proposed. ML techniques were also applied to DD experiments for a variety of tasks, ranging from signal classification to fast likelihood evaluation [31–36]. Ref. [32] utilizes a semi-unsupervised deep neural network comprising a pretrained convolutional neural network (CNN) and a VAE in order to detect the presence of excess nuclear recoils above the expected background in DD experiments.

The established approach to the detection of a new physics signal in DD experiment with dual dual-phase target is a likelihood-based test with an assumed asymptotic distribution [9], with the likelihood a function of the so-called “corrected” S1 and S2 signals (cS1 and cS2, respectively). By using neural networks that are trained on high-dimensional representations of detector events, we show in this paper that it is possible to infer the relevant properties (energy distribution, type of recoil) from detector-level readouts, without the approximation and loss of information incurred in the usual cS1, cS2 compression. This opens the door to the possibility of an end-to-end inference approach that is fully simulation-based, including all necessary corrections and cuts that are traditionally done in the analysis and inference chain, a process which takes up a significant fraction of analysis time in current-generation detectors. This approach relies however, on the availability of accurate and faithful simulations: real detectors and backgrounds are usually more complex and/or feature unexpected characteristics that deviate from simulations. Data-driven calibration and adversarial training techniques can help mitigate such systematic differences, improving robustness against these biases – something we plan to explore in future works.

Subject to the above caveat, the aim of this paper is to demonstrate the capability of a deep learning pipeline to detect the presence of an ‘anomalous’ signal above a known (from simulations) background in DARWIN, without explicit modeling of the likelihood nor of the physics underlying the anomaly (i.e., without assuming a specific dark matter model). In this sense, our analysis is model-independent, that is, agnostic to any specific new physics model. We achieve this by training an anomaly detector on event-by-event simulated detector response quanta using the DARWIN simulation pipeline, and by constructing an anomaly score designed to maximize the sensitivity to rejecting the background-only hypothesis. The choice of DARWIN as a case study is motivated by the availability of sufficiently mature and complete detector simulations, which is not yet the case for XLZD. Of course, the general approach is applicable to future detectors, once their design and simulation pipeline are settled. Application of this approach to existing detectors would require refinement to account for rare and/or unforeseen backgrounds or detector effects that may not be simulated correctly. Since this paper focuses on demonstrating the overall methodology, we leave exploration of such issues to future investigations.

This paper is structured as follows. In Sect. 2 we briefly introduce the design of the DARWIN detector, we describe the data structure used to train the model, as well as the simulations that were employed to this end. In Sect. 3 we explain the aim of the analysis, the methodology employed and its novelty. We also present the detailed simulation pipeline adopted for the study, the split between training and validation sets and the training procedure. In Sect. 4, we validate our approach by determining the sensitivity of DARWIN to rejecting the background-only null-hypothesis in the presence of a simulated injection of a WIMP signal. We then conclude in Sect. 5.

## Experiment design and data simulation

### The DARWIN detector design

DARWIN is conceived as a multi-ton, dual-phase liquid xenon time-projection chamber (TPC) designed to push DD sensitivity to the verge of the astrophysical neutrino floor [37]. The reference design holds $\sim 50\,$t of xenon, with about $40\,$t active, inside a $2.6\;\text {m}\times 2.6\;\text {m}$ cylindrical TPC; prompt VUV scintillation (S1) and proportional electroluminescence (S2) are captured by matched top and bottom arrays of ultra–low–background photomultipliers (PMTs) or silicon photomultiplier (SiPM) tiles, providing sub–keV thresholds and event–by–event electron vs nuclear recoil discrimination. The large homogeneous target, excellent self–shielding and simultaneous light–and–charge readout make large TPC chambers versatile platforms for dark matter, neutrino and rare decay physics [8].

The TPC design is suspended in a double–walled low–radioactivity cryostat and immersed in an instrumented water tank that serves both as a passive $\gamma / n$ shield and an active Cherenkov muon veto. A uniform drift field of the order of 0.5 kV cm$^{-1}$ is generated inside the TPC, enabling electrons to traverse the full 2.6 m height. This long-drift capability- as well as cryogenics, purification, and DAQ concepts, has been validated in the Xenoscope vertical demonstrator and related optical simulation test–stands [38], as well as a second large scale demonstrator called PANCAKE [39].

In 2024 the DARWIN, LZ and XENONnT collaborations unified their efforts in the next-generation XLZD programme [10], which scales the dual–phase concept to 60–$80\,$t of active xenon while retaining the core detector architecture. DARWIN’s hardware prototypes and simulation tools remain the principal testbeds for XLZD component development and the waveform-level analysis showcased here. Consequently, the study performed in this paper adopts the original 40 t DARWIN geometry when generating simulated S1/S2 events, with the ML methodology and data analysis pipeline having direct application to any future XLZD-type detector.

### Generation of simulated events

**Fig. 1:**
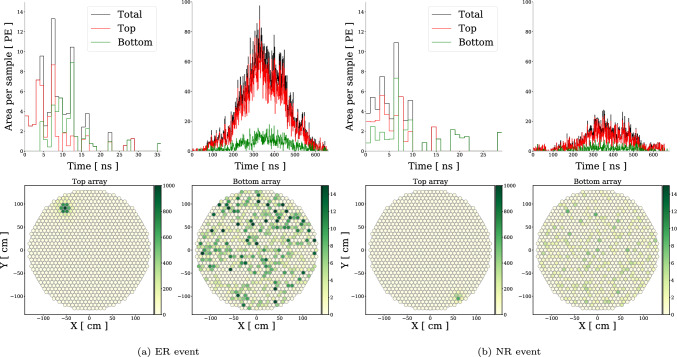
Example of simulated detector observables of an electron recoil (ER) **a** and nuclear recoil (NR) **b** event in DARWIN. **Top**: Number of S1 (left sub-panel) and S2 (right sub-panel) photoelectrons (PE) as a function of time after initial S1 triggering. Red (green) denotes observation in the top (bottom) PMT array. The black curves are the total S1 + S2 and are used for training the neural networks. **Bottom**: Top and bottom S2 PMT deposit spatial pattern. The color bar indicates the PMT hit count. These data are used to train the neural networks

Our simulation-based pipeline is reliant on the quality of the simulations adopted. For this reason, we use state-of-the-art simulations tailored to the DARWIN design. We use the Geant4 transport code [40] within the DARWIN-Geant4 framework [41] to handle the tracking of particles within a rendering of the detector geometry. The Noble Element Simulation Technique (NEST) v2.3.12 [42] handles the microphysics of how particles interact with the active xenon volume. NEST provides a robust and well-established framework that simulates the atomic and nuclear physics involved in energy deposition and the corresponding response of the detector, and generates the light and charge yields for each type of interaction within the detector. These simulated light and charge yields are compared and calibrated against previous xenon experiments, see Ref. [9] for details. Full signal propagation and observable readout within the TPC volume that produced the simulated waveforms and PMT hit-patterns were produced by custom-written detector simulation code based on the Tray [43] architecture.

Any WIMP search relies on distinguishing between background events and the WIMP-induced signal. We therefore need our deep learning pipeline to learn to characterize the background distribution. The majority of background at DARWIN will be electron recoil (ER) events originating from various terrestrial and cosmogenic sources, while nuclear recoil (NR) backgrounds remain in the form of irreducible cosmogenic neutrinos and sub-dominant radiogenic neutrons [41, 44], which must be included as part of the background simulation. WIMPs of mass $\mathcal {O}(>1)$ GeV deposit their energy into the detector via NR events.

We describe the background simulations used in this study in Sect. Appendix A, and give here only a concise summary. For each type of background (ER and NR), events with uniformly distributed recoil energies were simulated in the range 1–100 keV. The simulations include detector response effects (including electron-ion recombination, electron drift, and photon-collection efficiency), which transform the raw energy deposition from the initial particle interaction into the observable signals in the detector.^1^

For our analysis, we follow the approach taken in Ref. [32], and adopt as description of the TPC data the total S1 + S2 waveforms (i.e, signal as a function of time, summed over all individual PMTs), as well as the top and bottom S2 PMT hit pattern readout.^2^ We use the total waveforms (as opposed to the PMT-specific waveform) in order to reduce the dimensionality and complexity of the data vector provided to the neural networks. To exploit the detector readout data in even more fundamental form, one should adopt a model capable of learning a representation of the PMT responses from the entire PMT array in the temporal domain [46, 47] – something the method in this work is unable to scale to. Modern developments in Transformer or graph neural network architectures could potentially be used for handling time-domain individual PMT readouts [48–50]. In order to meet this challenge however, we plan to utilize the Rotary Masked Autoencoder of Ref. [51].

In Fig. 1 we show an example of the data used to train the neural networks. Events are simulated in a fiducial detector volume (FV) of 31.5 t, chosen to optimize the detection of rare NR while minimizing ER background interference towards the boundaries of the bulk xenon, as well as other factors [9]. The simulations are realized with a drift field of 200.0 V/cm, registering events when at least 4 photons are detected within a 200-nanosecond window (referred to as a ‘4-fold coincidence’, or N4T200). We do not utilize spatial reconstruction to provide a further fiducialization cut. Work is being done in this direction at XENON, see for example Ref. [52].

## Methodology

In this section, we first provide an overview of the objective of this study, followed by a concise description of the analysis methodology, which highlights the novelty of the approach. The architectural details as well as hyperparameters of the VAE and classifier used in this study are detailed in Appendix B.

### Simulation-based anomaly detection

The objective of this study is to demonstrate the potential of a deep learning pipeline to detect a WIMP-like signal above known simulated backgrounds in a semi-supervised fashion. This is complementary to the traditional likelihood-based method, as it offers several potential advantages: first, our approach makes fuller use of the information contained in the PMT readout data, thus avoiding the information loss that compression into summary statistics (such as cS1/cS2) inevitably incurs; secondly, it can incorporate in the pipeline any effect that can be faithfully simulated in the mock data. This means that the impact of nuisance parameters can be accounted for by simply including their sampling within the generation of training data. Finally, our approach does not rely on approximations to the likelihood, nor to a model-specific form of the WIMP-signal, therefore being more general and model-agnostic.

Our aim is to train a suitable neural network to identify anomalous signals – i.e., any event that can be distinguished statistically from the simulated ER and NR background distribution. This involves the computation of an ‘anomaly score’, *TS*, obtained from the combined loss distribution and classification output of a neural anomaly detector. The anomaly score is used to ascertain whether a collection of observed events $\textbf{X}_n = \{ {\textbf {x}}_1, {\textbf {x}}_2,\dots , {\textbf {x}}_n\},$ deviates from the background-only distribution. The null hypothesis, which we denote $\mathcal {H}_0$, is that the events ${\textbf {X}}_n$ are drawn from a distribution where no signal is present, i.e., compatible with the expected background.

The anomaly detector consists of two parts: a supervised binary classifier and a VAE. The classifier learns from training data to distinguish ER from NR events, whilst the VAE is trained solely on ER events^3^. After training, validation data (i.e., that the network has not been trained on) is given to the network, and its *TS* distribution obtained: events that deviated from background-like properties will manifest in the 1D space of the *TS* distribution as an excess over the background-only distribution. A simple 1D statistical test is then employed to reject the background-only hypothesis.

### Definition and distribution of the anomaly score

The anomaly score, *TS*, is defined as the weighted linear combination of the reconstruction loss from the VAE, or ‘ELBO’ (see Eq. B.2), and the classifier’s binary cross-entropy, $H_B$, so that larger values correspond to deviations from the null hypothesis:

1 $$\begin{aligned} TS&= (-\text {ELBO}) + RH_B \nonumber \\&= D_\text {KL}(q(\textbf{z} | \textbf{x}_\text {in}) || p(\textbf{z})) - \mathbb {E}_{q(\textbf{z}|\textbf{x}_{\text {in}})}[\log p_{\textbf{x}_{\text {in}}}(\textbf{x}_\text {D} | \textbf{z})] \end{aligned}$$

2 $$\begin{aligned}&\quad + R\, H_B(\textbf{x}_{\text {in}})\nonumber \\&= - \frac{1}{2} \beta \sum _{j=1}^{m}\left( 1+\log \left( {\sigma }_j^2\right) -\mu _j^2-\sigma _j^2\right) \nonumber \\&- \log \mathcal {N}_{\textbf{x}_{\text {in}}}( \textbf{x}_\text {D}, \text {diag}(\boldsymbol{\sigma }_\text {D})^2) -R \log \left( 1-p\left( \textbf{x}_{\text {in}}\right) \right) \;. \end{aligned}$$

The hyperparameter *R* controls the relative importance of the binary cross-entropy term, and its optimization is discussed in  Appendix C.

**Fig. 2:**
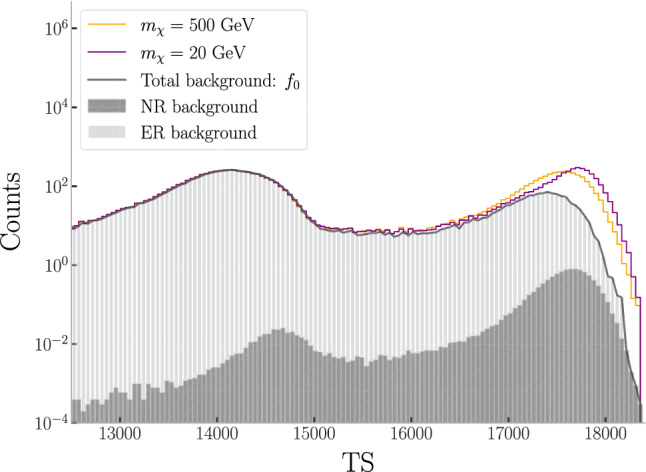
Distribution of the anomaly score *TS* from a pseudo-dataset used in this study. The stacked gray bars represent the *TS* distribution for the ER (light gray) and NR (dark gray) background. The colored lines are the distributions in *TS* after the injection of signal components for 20 and 500 GeV WIMPs, with a scattering cross-section of $\sigma _\chi = 10^{-46}$ cm$^2$ (a large value chosen for clarity of illustration). The binning is illustrative, as our sensitivity analysis is unbinned. The solid black line is the total background pdf $f_0$

In order to determine the *TS* distribution under $\mathcal {H}_0$, a set of $10^4$ ER and $10^4$ NR events are simulated according to their expected rates after trigger-level cuts, fiducialisation and signal region cuts, as given in Fig. 5 of Appendix A. In Fig. 2 we show a dataset comprised of each background component (dark/light grey histogram) as well as two injected WIMP signals (color curves) at a relatively large cross-section (for illustration purposes) in *TS* space, re-weighted to an exposure of 200 ty. The spectral dependence of the ELBO manifests in *TS* space, with anomalous events (in this case, WIMPs) being mapped to larger *TS* values than the background. We therefore observe two bumps in the *TS* distribution of the NR and ER backgrounds corresponding to the classifier’s prediction. ER events that present with higher *TS* values typically have lower energies, as would make qualitative sense due to low-energy ER being indistinguishable from NR. In Appendix D, we demonstrate that the VAE non-trivially encodes the spectral energy information of all events (both NR and ER), despite the VAE having been trained only on ER events.

### Neural networks training and validation

The neural networks are trained on vectorized formats: [S1WaveformTotal, S2WaveformTotal, S2Patter ns], with a total size of 3835. The waveform and hit pattern data provide information about each event, making it possible for the neural anomaly detector to learn complex features pertaining to the class of the event (ER vs NR) as well as the different spectral dependency of each class (see Appendix D and Appendix E for further details).

We generate training data sets consisting of an even sample of $2\times 10^4$ ER and NR events with true recoil energies uniformly distributed in $E_R\in [1,100]$ keV, with 30% being kept aside for validation. The average training time per epoch is $\sim$ 1 s for the VAE ($\sim 40$ seconds total training time) and $\sim 0.8$ seconds for the classifier ($\sim 8$ seconds total training time) on an NVIDIA A100-PCIE-40GB GPU. Testing times event-by event are of the order of ms.

### Null hypothesis test

In order to test for the presence of an anomalous bump (due to anomalous, non-background-like events) in the *TS* distribution, we define an unbinned 1D likelihood for the background probability distribution function (pdf), $f_0$, called the ‘extended Poisson’ [54]:

3 $$\begin{aligned} \mathcal {L}(\textbf{TS}|\mathcal {H}_0 ) = \frac{e^{-B}}{N!}\prod _{i=1}^NB f_0\left( TS_i \right) \, . \end{aligned}$$

Here $\textbf{TS}$ denotes the vector of observed *TS* produced by the trained neural network for events labeled by *i* during a given exposure, while $B$ is the total expected number of background events and *N* is the number of observed events.

**Fig. 3:**
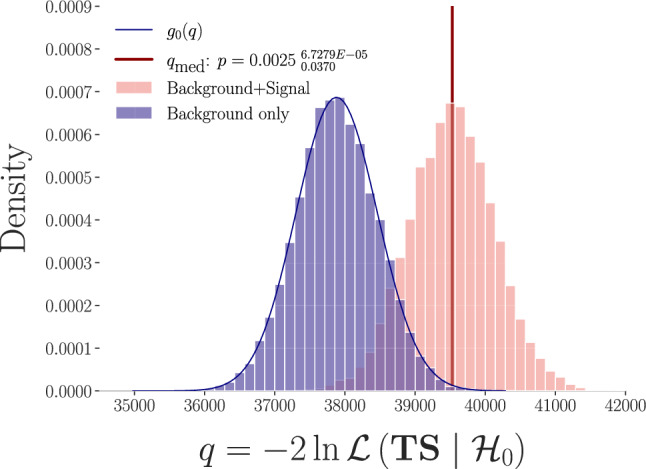
Distribution of $q=-2 \ln \mathcal {L}(\textbf{T S} \mid \mathcal {H}_0)$ from pseudodata generated under $\mathcal {H}_0$ (blue) and with an injected dark matter (WIMP) signal with $\sigma _\text {SI}=6.5\times 10^{-48}$ cm$^2$ and $m_\chi = 50$ GeV (pink), which yields a median sensitivity of $\sim 3\sigma$ at 200ty exposure. We also display as a blue line the kernel density estimate (KDE) used to evaluate the integral in Eq. (4). The red vertical line denotes $q_\text {med}$

We take as a test statistic the distribution of $q = -2\ln \mathcal {L}$, formalizing $\mathcal {H}_0$ as the asymptotic distribution of *q* after simulating $\sim 10^4$ experiments, each with an exposure of 200 ty, using pseudo-datasets comprised solely of background events, where the number of events per experiment is sampled from a Poisson with expectation value *B*, leading to a number of events per experiment $\sim \mathcal {O}(6.5\times 10^3)$. This distribution of *q* is shown in blue in Fig. 3. Any upward fluctuation of the negative log-likelihood denotes a departure from the background-only hypothesis by construction. The distribution of *q* from another $10^4$ simulated experiments including an injected WIMP signal at a fixed benchmark of $\sigma =6.5\times 10^{-48}$cm$^2$, $m_\chi = 50$ GeV is shown in pink, while the median significance $q_\text {med}$ (i.e., the median $p-$value for which one can reject $\mathcal {H}_0$ in the presence of a signal, calculated over a collection of pseudo-datasets [55]) is denoted by the vertical red line. The median sensitivity is the $p-$value to reject $\mathcal {H}_0$ corresponding to $q_\text {med}$:

4 $$\begin{aligned} p_\text {med} = \int _{q_\text {med}}^\infty \,dq\; g_0\left( q\right) \;, \end{aligned}$$

where $g_0(q)$ is the distribution of *q* under the null hypothesis.

**Fig. 4:**
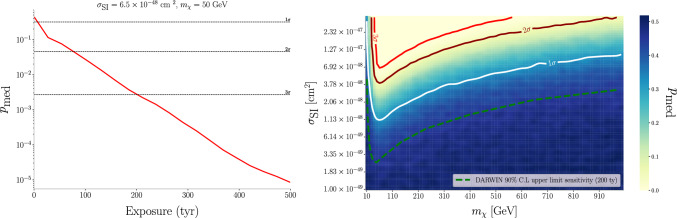
**Left:** Median sensitivity to reject the background-only hypothesis as a function of detector exposure at the benchmark $\sigma _{\textrm{SI}}=6.5 \times 10^{-48} \mathrm {~cm}^2, m_\chi =50\, \textrm{GeV}$. Thresholds of 1,2 and $3\sigma$ decision boundaries are shown as black horizontal dashed lines. **Right:** Median sensitivity in the $m_\chi$, $\sigma _\text {Si}$ plane from the anomaly detection pipeline (exposure of 200 ty), with contours at 1, 2 and $3\sigma$ (solid lines). For qualitative comparison, the WIMP-model dependent DARWIN 90% C.L. median upper limit sensitivity is shown as the green dashed line

## Results

In this section, we present the results from our approach on simulated data. For this analysis, the ER and NR background distributions have been re-weighted to their expected values using the background benchmarks from  Appendix A. The median sensitivity to reject $\mathcal {H}_0$ as a function of exposure is shown as the red line in Fig. 4 (left panel) for the WIMP benchmark adopted in Fig. 3 ($\sigma _\text {SI} = 6.5\times 10^{-48}$ cm$^2$, $m_\chi = 50$ GeV).

The right panel of Fig. 4 shows the median sensitivity in the canonical 2D WIMP parameter space for a fixed exposure of 200 ty. We plot the median sensitivity as a color gradient, indicating contours corresponding to 1, 2 and $3\sigma$ median sensitivity. For qualitative comparison only, we display the 2016 median DARWIN 90% C.L. upper limit sensitivity as a green dashed curve [8]. It is important to note that this 90% C.L upper limit sensitivity is not directly comparable to the background rejection test in our pipeline, as these are two fundamentally different statistical tests: the 90% C.L upper limit sensitivity is model-dependent (as the WIMP signal is specific for a given model), whilst the anomaly detection method is agnostic to the WIMP physics, as the neural networks were only trained on samples indicative of a background-only dataset, with no information about WIMP-like events. Hence, whilst the background rejection *p*-value we present is a somewhat ‘stronger’ statistical claim (in that it is model-independent), we find (as expected) that an upper limit in the presence of an explicit alternative WIMP model is significantly more constraining.

## Conclusions

This study presents the foundation for a deep learning analysis pipeline to perform anomaly detection in next next-generation dark matter direction detection experiment – in this case, the DARWIN design. The proposed methodology provides a prototype for future developments in statistical inference in rare physics searches with xenon-based TPCs, and promises to extract maximal information from the high-dimensional event data produced by TPC experiments. This is particularly critical given the current challenges faced by modern TPC experiments, where a substantial portion of analysis time is devoted to tuning optimal cuts and corrections for high-level, compressed summary observables.

The method in this paper presents an anomaly-aware machine learning technique that leverages deep learning to conduct a background rejection task. We use a neural network architecture consisting of an unsupervised VAE and a fully connected classifier that extracts relevant event-by-event features (including energy information) from PMT hit pattern data and total S1 and S2 waveforms. We find that the neural anomaly detector achieves sensitivity to reject $\mathcal {H}_0$ at the order of $3\sigma$ after $\sim 200$ ty for a WIMP benchmark of $\sigma _{\textrm{SI}}=6.5 \times 10^{-48} \mathrm {~cm}^2, m_\chi =50\, \textrm{GeV}$.

A model-independent anomaly detection can serve as a ‘first pass’ analysis, assessing if there is any data that is not consistent with the background-only expectation, before moving on to a more sensitive model-dependent search (e.g., via likelihood ratio). Whilst we have validated our pipeline in the context of a canonically interacting WIMP, the machinery remains identical for any new physics search. This makes the development and deployment of these types of analyses an important addition to the standard statistical pipeline.

As is always the case for simulation-based analyses, the neural networks could be subject to missing or misinterpreting key underlying features or stochastically of real data should the simulations be incomplete or otherwise imperfect [56, 57]. To mitigate this risk, one could expand the pipeline to include fine-tuning the models on calibration data in the training of the neural network, thereby complementing simulated events with actual observations. A large computational effort is currently being directed toward folding in calibration information into the derivation of the high-level cS1/cS2 statistics, something that would be complemented by our approach: a neural network-based analysis pipeline can alleviate the computational burden as it bypasses the need for these corrections. However, care must be taken with uncertainties due to specification of the recoil energy of events, especially at lower energy thresholds [58, 59]. This type of issue could be circumvented with unsupervised anomaly detector networks that have integrated domain adaptation between simulated source data and target calibration [60]. Investigation of these types of models will be the subject of future work.

Given the simulation-rich environment at DARWIN and in the future, XLZD, we plan to leverage this approach, including multi-scatter classification, energy and position reconstruction, circumventing the need for traditional detector fiducialisation or signal region definition. Other architecture developments will be aimed at handling high-dimensional temporal PMT data, accidental coincidence, and surface events background discrimination, as well as inter-ER background classification.

## Figures and Tables

**Fig. 5:**
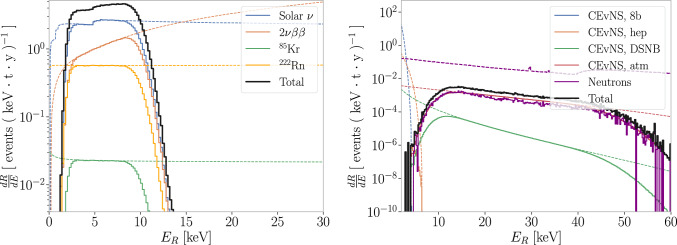
Benchmark DARWIN background differential recoil rate spectra considered in this analysis, before (dashed lines) and after (solid lines) detector-level SR, fiducialization and threshold cuts. The total background contributions are shown by black solid lines. **Left:** ER backgrounds originating from low-energy solar neutrinos, two-neutrino double-beta decays of $^{136}$Xe and intrinsic backgrounds from $^{85}$Kr and $^{222}$Rn. **Right:** NR background contributions, produced by coherent neutrino-nucleus scattering sources: solar neutrinos originating from $^8$B and from the helium-proton reaction, atmospheric neutrinos, the diffuse supernova neutrino background, and radiogenic neutrons from the detector. The mean integrated rates are given in Table 1 for each background component

**Fig. 6:**
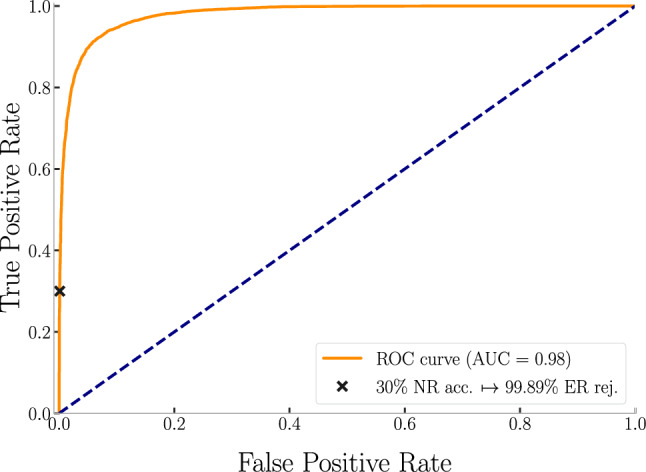
Receiver operating characteristic (ROC) curve of the supervised classifier trained to discriminate ER vs NR events, evaluated on a testing set consisting of an evenly mixed sample of $10^4$ NR and $10^4$ ER events. The area under the curve (AUC) is 0.98. The dashed blue lines indicate a random classifier. The black cross denotes the false positive rate (FPR) at a true positive rate (TPR) of 0.3, corresponding to the ER rejection capability of the classifier when the NR acceptance is 30%

**Fig. 7:**
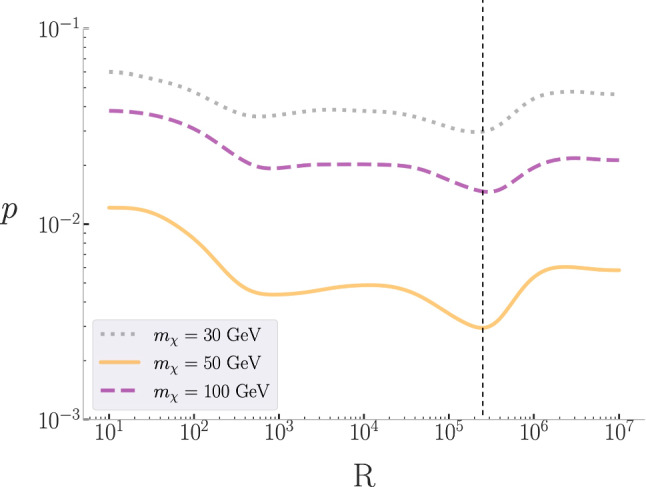
optimization of the hyperparameter *R* that controls the contribution of the supervised classifier in the determination of the anomaly score *TS*. The $p-$value to reject $\mathcal {H}_0$ is given as a function of *R* for three benchmark WIMP masses at fixed exposure of 200 ty and cross-section $\sigma _\text {SI} = 6.5\times 10^{-48}$ cm$^2$. As the scattering cross-section merely rescales the median sensitivity, the choice of *R* and cut value are insensitive to it. The optimal value for $m_\chi =50$ GeV is $R=2.5\times 10^5$, shown by the vertical dashed line. The variation in the location of optimal *R* value is small for other mass benchmarks

**Fig. 8:**
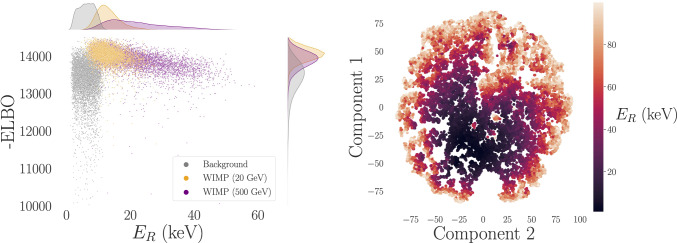
**Left:** ELBO values as a function of ground truth recoil energy $E_R$ for a validation set of events: total (ER+NR) background (gray) and events from two WIMP benchmarks. The 1D marginals of the ELBO and $E_R$ are also shown. Distributions are normalized densities for illustration purposes. The separation in the 2D space shows that spectral information has been encoded within the ELBO. **Right:** 2D tSNE of the trained VAE’s 128-dimensional latent space for a validation sample of ER events with true recoil energies in the range $E_R\in [1-100]$. The color scale represents ground-truth recoil energy $E_R$ of the events. The non-trivial latent structure in $E_R$ confirms that the model has learned spectral information

**Fig. 9:**
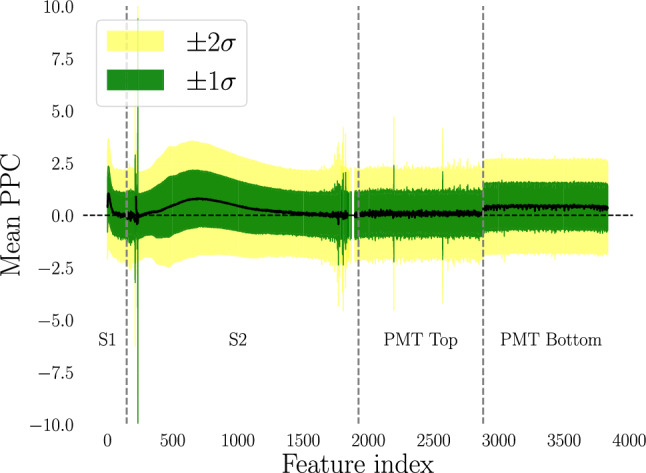
Posterior predictive checks performed on $10^4$ samples from the latent space of the trained VAE. A perfect VAE would produce a PPC of zero for all feature indices. The black curve is the mean PPC from Eqn. (E.4), with $\pm 1\sigma$ and $2\sigma$ estimates shown as green and yellow bands, respectively. Each feature index corresponds to an element of the input data vector ${\textbf {x}}_\text {in}$. The vertical gray dashed lines demarcate the subdivision into the S1/S2 wave-forms and S2 PMT Top and PMT Bottom hit patterns

## Data Availability

This manuscript has no associated code/software. [Author’s comment: Data sharing not applicable to this article as no datasets were generated or analysed during the current study.]

## Background modeling

In this section, we detail the background modeling of this study. The different sources of ER and NR backgrounds relevant to DARWIN are described in Refs. [8, 41, 44].

**Table 1:** Summary of mean background event rates after detector-level SR, fiducialization and threshold cuts

Background event rates	
	$$\overline{\textrm{Rate}}\;\big [(\textrm{ty})^{-1}\big ]$$
ER intrinsic: $^{136}$Xe $(2\nu \beta \beta )$	9.4
ER intrinsic: $^{222}$Rn	4.5
ER intrinsic: $^{85}$Kr	0.18
ER solar neutrinos	20.0
NR solar CEvNS	$5.3\times 10^{-4}$
NR atmospheric CEvNS	$2.6\times 10^{-2}$
NR radiogenic neutrons	$2.2\times 10^{-3}$

The background contributions in DARWIN can be categorized into external and intrinsic backgrounds: external backgrounds include gamma-rays and neutrons originating from radioactive decays or interactions outside of the target volume. These can be significantly reduced by target fiducialization due to the high density of liquid xenon. Intrinsic backgrounds, on the other hand, are uniformly distributed in the target region and cannot be reduced by fiducialization.^4^

The background is obtained after detector-level cuts, including the finite energy threshold of the detector, the fiducial region and signal region (SR) cuts on the combined energy scale (CES), and an estimate of the true deposited recoil energy, $E_R$. For this analysis, we adopt a standard value of 31.5 t [8], using an estimated location in the detector for fiducialization cuts. Furthermore, given that the spectral information of all relevant backgrounds is not currently fully known, we apply a [2–10] keVee cut on the CES of each event in line with previous studies [44]. This leaves ERs with a ground truth $E_R$ between $\sim$[2–14] keV and NRs between $\sim$[2–60] keV, which are displayed in Fig. 5. A further assumption we make is that multi-scatter events are fully vetoed. Thus, the analysis presented in this work assumes 100% single-scatter selection efficiency.^5^ Each background contribution has an expected rate as shown in Table 1.

**ER backgrounds:** Solar neutrinos produced through the proton–proton (*pp*) fusion process and the subsequent beryllium-7 ($^{7}$Be) reaction in the Sun are the dominant source of ER background for dark matter searches beyond the ton-scale. This is because of their relatively low energies and high abundance, along with the fact that their contribution cannot be reduced by target purification, fiducialization, nor single-scatter selection. Intrinsic backgrounds, including contributions from isotopes such as ^85^Kr, a beta-emitter present in natural krypton, and ^222^Rn, are included. These intrinsic backgrounds are uniformly distributed in the target due to the chemical inertness of noble gases. Two-neutrino double-beta decays ($2\nu \beta \beta$) of ^136^Xe yield a background that steeply rises with recoil energy. Finally, ER backgrounds originating from $\gamma$-rays from radioactive contamination in the cryostat and detector materials are reduced to negligible amounts by target fiducialization, hence we neglect them here [44]. The differential energy spectra of the above four ER background contributions are shown in Fig. 5 (left panel), both before and after detector-level event cuts.

**NR backgrounds:** Radiogenic neutrons emitted from the detector’s materials, particularly from light PTFE used as insulator and light reflector, and photosensors made from various materials constitute a primary source of NR background.^6^ Fiducialization of the detector volume serves as the primary detector-level cut on the radiogenic neutrons, which extensive Geant4 simulations indicate as interacting primarily near the detector walls. Furthermore, neutrons can scatter multiple times within the detector volume. A veto on such multi-scatter events, determined from the S2 area distribution, is implemented with an assumed 100% efficiency.

The neutron background contributes more at larger (10–50 keV) recoil energies relative to the significantly more perilous other NR backgrounds, namely, coherent elastic neutrino-nucleus scattering (CEvNS) [44]. $\,^8$B solar neutrinos are primarily responsible for a steep rise in background events at low recoil energy, hindering the detection of low-mass WIMPs (5–8 GeV). This background is difficult to distinguish from WIMP signals and represents a limit on sensitivity [62], at least for non-directional DD experiments.

At higher recoil energies, the main CEvNS background is from atmospheric neutrinos (atm), with smaller contributions from solar neutrinos from the helium-proton reaction (hep) and the diffuse supernova neutrino background (DSNB) [41, 63]. The spectra of NR backgrounds considered in this study are shown in Fig. 5 (right panel).

Lastly, as noted in Sect. 3.1, by propagating nuisance-parameter variations through the simulation and training on the resulting samples, the neural network effectively learns to marginalize over these uncertainties. For the generation of the PMT and waveform data used in this study, such nuisance parameters included within the $\texttt {Tray}$ framework include PMT-quantum efficiencies, Light collection efficiency (LCE) uncertainties, systematic uncertainties associated with the S1/S2 reconstruction, as well as single photo-electron (SPE) area response.

## Neural network architectures

In this section, we describe the components of the neural networks in detail. All neural networks are trained with Tensorflow v2.15.0 [64].

### Variational autoencoder (VAE)

The goal of an autoencoder is to learn a compressed representation (encoding) of the input data, and then reconstruct the input data from this encoding [65, 66]. Autoencoders encompass three primary components: an encoder, a latent space, and a decoder. The encoder reduces the input data vectors ${\textbf {x}}_\text {in} \in \mathbb {R}^n$ into a lower-dimensional latent space representation ${\textbf {z}} \in \mathbb {R}^m$ (with $m\ll n$) through a transformation ${\textbf {z}}=f({\textbf {x}})$. The decoder then reconstructs the input from this compressed form, aiming to produce an output ${\textbf {x}}_D =g({\textbf {z}})$ as close to the original ${\textbf {x}}_\text {in}$ as possible. A reconstruction loss function, quantifying the difference between ${\textbf {x}}_\text {in}$ and ${\textbf {x}}_D$, is optimized during training.

Variational Autoencoders (VAEs) extend this concept by introducing a probabilistic approach to the encoding process. Unlike standard autoencoders, the encoder in a VAE maps input data to a probability distribution characterized by mean $\mu$ and variance $\sigma ^2$, essentially transforming the encoder’s output into the parameters of a Gaussian distribution:

$$ f({\textbf {x}}_\text {in}) \rightarrow q({\textbf {z}} \mid {\textbf {x}}_\text {in}) = \mathcal {N}_{\textbf {z}}\left( {\boldsymbol{\mu }, \text {diag}(\boldsymbol{\sigma }^2)}\right) \,. $$

The decoder, now governed by $g(\textbf{z})\rightarrow p(\textbf{x}_D|\textbf{z})$, is a probabilistic distribution that reconstructs data from sampled points in this probabilistic latent space. When $\textbf{x}_D$ are real vectors, $p(\textbf{x}_D|\textbf{z})$ is taken to be a multidimensional normal distribution with diagonal covariant structure^7^ [68]:

B.1 $$\begin{aligned} p(\textbf{x}_D|\textbf{z}) = \mathcal {N}_{\textbf{x}_{\text {in}}}( \textbf{x}_\text {D}, \text {diag}(\boldsymbol{\sigma }_\text {D}^2))\;. \end{aligned}$$

The VAE is trained via stochastic gradient descent by maximizing the loss function given by the so-called ‘evidence lower bound’ or $\text {ELBO}$ [68]:

B.2 $$\begin{aligned} \quad \text {ELBO}&= \mathbb {E}_{q(\textbf{z}|\textbf{x}_{\text {in}})}[\log p_{\textbf{x}_{\text {in}}}(\textbf{x}_\text {D} | \textbf{z})] - D_\text {KL}(q(\textbf{z} | \textbf{x}_\text {in}) || p(\textbf{z})) \nonumber \\&=\frac{1}{L} \sum _{l=1}^{L} \log \mathcal {N}_{\textbf{x}_{\text {in}, l}}( \textbf{x}^\text {D}_l, \text {diag}(\boldsymbol{\sigma }^\text {D}_{l})^2)\nonumber \\&\quad \quad \quad + \frac{1}{2} \beta \sum _{j=1}^{m}\left( 1+\log \left( {\sigma }_j^2\right) -\mu _j^2-\sigma _j^2\right) \;, \end{aligned}$$

where *m* is the dimensionality of the latent space (number of independent Gaussians), the expectation is under the distribution $q({\textbf {z}} \mid {\textbf {x}}_{\text {in}})$ and the data are batched into batches of size *L*. The coefficient $\beta$ in the Kullback–Leibler (KL) term balances its regularization strength [69], with a higher $\beta$ value ensuring that the encoded representations are closer to the prior, $p({\textbf {z}})$, taken to be a standard multivariate Gaussian, $\mathcal {N}(\textbf{0}_m,\textbf{1}_m)$.

The VAE architecture used in this study was selected after hyperparameter optimization on validation datasets withheld from training, and inspired by previously successful architectures in similar settings, in particular Ref. [32]. It consists of an encoder that takes vectorized data inputs $\textbf{x}_\text {in}$ (see Sect. 3.3) in batches of size $L=10$ and processes it through two dense (i.e., fully-connected) layers with 2000 and 500 units respectively. The latent space dimension is $m=128$. The decoder has a dual-network structure. Both networks within the decoder begin with an input of shape 128, and process it through dense layers of 500 and 2000 units, culminating in two output layers $\textbf{x}_D$ and $\log \boldsymbol{\sigma }^2$ with shape matching $\textbf{x}_\text {in}$. The architecture is summarized in Table 2. We note that this architecture does not scale for use on raw time series PMT readout data, given that dense, fully connected neural networks are not optimal for the sparsity one would expect from such data (leading to optimization issues and computational inefficiency). Therefore, a more suitable architecture, such as the one presented in Ref. [70], would be needed to use raw data as input.

For training, we use an Adamax optimizer with a learning rate of $0.5 \times 10^{-3}$. The entire training regimen is set to run for 30 epochs, with an optimized $\beta$ value of 10 (via uniform hyperparameter scans). Validation tests of the so-obtained latent space representation are presented in  Appendix E.

**Table 2:** Summary of the VAE architecture and optimal hyperparameters. All dense layers have ReLU activations except for the linear input and output.

**Variational autoencoder architecture**	
Latent dimension, *m*	128
$\beta$	10
Encoder	Input Layer: Shape: 3835
	Dense Layer: 2000 units
	Dense Layer: 500 units
	Dense Layer: $2 \times$m
Decoder	Input Layer: Shape: *m*
	Dense Layer: 500 units ($\times 2$)
	Dense Layer: 2000 units ($\times 2$)
	Dense Layer: 3835 units ($\times 2$)
Optimizer	Adamax, Learning Rate: 0.0005
Training epochs	30

### Supervised ER vs NR classifier

**Table 3:** Summary of the neural network classifier’s architecture and optimal hyperparameters. The activation function of all layers is ReLU, with the output being a sigmoid

**Classifier Architecture**	
Input Shape	Data Shape (3835)
Layers	Dense Layer: 256 units
	Dense Layer: 64 units
	Dense Layer: 16 units
	Output Layer: 1 unit
Optimizer	Adam, Learning Rate: 0.01
Training Epochs	5

The second component of the anomaly detector pipeline is a simple multi-layer perceptron (MLP) feed-forward neural network [71], whose architecture details are listed in Table 3. The classifier’s task is to perform a binary classification between ER (0 output value) and NR (output value of 1) events. The MLP is trained by minimizing the standard binary cross-entropy loss:

B.3 $$\begin{aligned} H_B=-\frac{1}{L} \sum _{i=1}^L \log \left( 1-p\left( \textbf{x}_{\text {in}}\right) \right) \end{aligned}$$

where $$ L = 10$$ is the number of samples in the batch, and $$p\left( \textbf{x}_{\text {in}}\right) $$ is the predicted class probability for each sample extracted from the sigmoid output of the MLP. The architecture details of this classifier are listed in Table 3.

In Fig. 6 we show the receiver operating characteristic curve (ROC) for a test set of $10^4$ ER and NR events. We observe an area under the curve (AUC) of 0.98. We compare our classifier’s performance with the 99.98% ER rejection obtained in previous DARWIN sensitivity studies [8, 9]. Such a large ER rejection probability is intended to mitigate leakage of ER’s into the WIMP NR signal region, but it comes at the expense of a lower NR acceptance, which is estimated at 30% in Ref. [9]. The false positive rate (FPR) for our classifier (which corresponds to ER leakage) at a true positive rate (TPR) of 0.3 (correct NR classification), is denoted by the black cross in Fig. 6, and is 0.11%, corresponding to 99.89% ER rejection. We note that the standard approach uses the assumption of Gaussianity for cS1 and cS2 to mitigate ER leakage. Our classifier, however, makes no such assumption as it operates on an event-by-event basis, and hence any misclassified events will simply modify the distribution of the anomaly score, see Sec. 4. We also tried modifications of the loss function in Eq. (B.3) to optimize the false positive rate (i.e., minimize the number of mis-classified ER). Whilst this indeed was successful, the number of mis-classified NRs also increased, leading to a net zero effect in the overall anomaly score presented in Eqn. (2). Finally, we note that the choice of architecture used to perform the event-by-event ER/NR classification is largely irrelevant, as was seen in Ref. [36], where comparable performance was observed from MLPs, transformers, and boosted decision trees.

## Optimization of the supervised contribution

The optimization of the hyperparameter *R* in Eq. (2) is a choice to be made at the time of analysis, in order to maximize the observation of any anomalous *TS* component, if it exists. To demonstrate this, we perform a scan over a range of logarithmically spaced *R* values $R\in [1,10^7]$ at fixed signal injection benchmarks corresponding to WIMP masses of 30, 50 and 100 GeV at an exposure of 200ty. These values of the WIMP mass were chosen in order to vary the spectral dependence of the induced WIMP signal. We show the median sensitivity, defined in Eq. (4), in Fig. 7, as a function of *R*, for the three benchmarks. A smaller $p-$value means better anomaly awareness and higher power to reject $\mathcal {H}_0$ in the presence of a signal, and thus *R* should be chosen to minimize this value. We conduct this test at a cross-section that yields a background rejection *p*-value of at least $\sim 3\sigma$ for $m_\chi = 50$ GeV, so as to have ample statistics to perform the test for all three mass benchmarks.

We observe that the spectral dependence of the anomaly function *TS* entering through the ELBO as observed in Fig. 8 does not affect the dependence of the optimal *R* value, which lies at $\sim 2.5\times 10^{5}$. The general variability of the $p-$value is much more pronounced for $m_\chi =50$ GeV due to DARWINs elevated sensitivity to this mass. We observe that for *R* values above $\sim 10^6$, the *p*-value exhibits a plateau, which we have checked persists for values $R>10^7$. This indicates that above this critical value of *R*, the influence of the VAE is vanishingly small.

The above results highlight the importance of taking a semi-supervised approach: the fact that the power to reject $\mathcal {H}_0$ is maximized for $R\ne 0, \infty$ shows explicitly the need for a combined supervised and unsupervised approach in order to maximize sensitivity to anomalous physics. In principle, *R* could be recast as a learnable parameter during training, although we chose to leave this to future study. For this work, we adopt an optimized *R* value of $R=2.5\times 10^5$.

Previous studies observed that classifiers can perform well as anomaly detectors (see, for example, Ref. [72]). An admixture of many supervised and/or unsupervised components could offer additional advantages, for example, by further exploiting the topological structure of events observed in the latent space. Indeed, Fig. 7 indicates non-triviality via the two observed local minima in the *R* dependence of the median sensitivity. We see that the latent data feature that is learned by the VAE was the event recoil energy, whilst the classifier learns the type of event. Both of these features are crucial to a new physics discovery, regardless of origin. It may then follow that other auxiliary models trained on the same and/or combinations/sets of prompt detector outputs may yield even better anomaly awareness. We leave this as an interesting question for future work in this domain.

## Spectral information encoding

The distribution of the ELBO from the VAE as a function of ground truth (simulated) event recoil energy $E_R$ is shown for a validation sample with true recoil energies in the range [1–100] keV in Fig. 8 (left panel). We plot the normalized spectral distributions in the space spanned by $E_R$ and ELBO for the total (ER+NR) background (gray) and for events generated by two WIMP masses, $m_\chi =20,500$ GeV (orange/magenta). We observe an interesting separation in the distributions, pointing to the fact that the VAE is capable, after training exclusively on background events, to distinguish the spectral distribution from WIMPs.

To visualize the latent representation of the data, we further show a 2-dimensional t-distributed stochastic neighbor embedding (tSNE) projection [73] of the 128-dimensional latent space of the VAE in Fig. 8 (right panel). The non-trivial structure of the latent space, even in a two-dimensional projection, demonstrates that spectral information has indeed been incorporated into the model.

## Validation of VAE representation

To validate the true low-dimensional latent features of the ER training data that were learned by the VAE, we carry out a standard benchmarking test known as a ‘posterior predictive check’ (PPC) [74, 75]. An ideal model will generatively produce samples that align with the target distribution, and therefore produce a $\text {PPC}\sim 0$. Given the one-dimensional nature of our data (after vectorisation), we adopt the following simple strategy: we generate *N* samples ${\tilde{\textbf{z}}} \sim \mathcal {N}({\boldsymbol{\mu }},\boldsymbol{\sigma }^2)$ from the latent space of the trained VAE and parsing them through the decoder, to obtain the predicted output, ${\tilde{\textbf{x}}}$. A separate test set $\textbf{x}_\text {test}$ that is withheld from training is then used to calculate the relative reconstruction error:

E.4 $$\begin{aligned} \text{ Mean }\left( \text {PPC}\right) ^{(j)}=\frac{1}{N}\sum _{i=1}^N\frac{\left( {{\tilde{x}}^{(j)}_{i}-{x}^{(j)}_{i_\text {test} }}\right) }{{{\sigma }^{(j)}_{\text{ test } }}}\;, \end{aligned}$$

where $\sigma _{\text {test}}^{(j)} = \sqrt{\frac{1}{N} \sum _{i=1}^N \left( x^{(j)}_{i_{\text {test}}} - \bar{x}^{(j)}_{\text {test}} \right) ^2}$ is the standard deviation of the distribution of test samples ${x}^{(j)}_{_\text {test} }$ for feature (vector column) $j=1, \dots , 3835$ and serves as a normalization factor. We use $N=10^4$.

We show the result of the PPC in Fig. 9 for all 3835 data features. For clarity, we demarcate with vertical lines the features corresponding to the S1 and S2 waveforms, as well as the top and bottom S2 PMT hit-patterns. We plot the mean PPC as a black curve with the $\pm 1,2\sigma$ uncertainties in green and yellow, respectively. While a perfect network would produce a PPC of zero for all features, we observe that our network’s output lies within $1\sigma$ of 0 for all features, indicating that it has learnt the underlying properties of the training data. The largest deviation from zero occurs for features at small pulse times for S1 and S2 waveforms (i.e., close to the start of the S1/S2 feature indices, indicated by the vertical lines). This is expected since most of the events used during training have a small or zero S1/S2 value at larger times (cf. Figure 1). Therefore, the network has fewer issues learning this degeneracy at large times and can reconstruct the corresponding features toward the ends of the S1/S2 feature index. This, however, can lead to larger variance in the PPC distribution of some features due to the model’s lack of reconstruction power in regions of degenerate zeros in the feature space, as are observed as spikes in the $1/2\sigma$ bands. We observe near-perfect reconstruction for the top S2 PMT array, but observe a slight, positive offset for the bottom PMT. We attribute this behavior to the bottom PMT displaying what is mostly uniform noise for the majority of ER events, as seen in Fig. 1. Hence, the values for which the VAE can optimize the ELBO are somewhat arbitrary and present as a systematic offset. The top PMT array, however, displays concentrated deposits that are well associated with the event properties and can therefore be learned more easily.

## Footnotes

1 Another form of background observed by XENONnT was anomalous events emanating from radioactivity in the TPC walls, referred to as ’surface’ events. We did not simulate such event realizations in this study, but work is being conducted to implement them with unsupervised veto models.

2 We note that not including the S1 top and bottom PMT hit-patterns decreases sensitivity to so-called ‘$\gamma$-X’ and ‘neutron-X’ eventsFootnote 2 continued as observed by XENON100 [45, 46]. Since we do not include such background events in this study, this has no bearing on our results here. Future developments will add both ‘$\gamma$-X’ and ‘neutron-X’ events to the background, and S1 PMT patterns to the input data.

3 An alternative approach (see, for example, the work of the LUX-ZEPLIN (LZ) collaboration in Ref. [53]) can be to train a VAE on aFootnote 3 continued representative sample of *all* event classes (comprising both ER and NR) as well as calibration data. This allows potentially anomalous events to be identified in the latent space of the autoencoder. Representation learning of this type can be useful in that it is one-sample and ’data-driven’, at the expense of sensitivity to an explicit null-hypothesis.

## Code Availability

This manuscript has no associated data. [Author’s comment: Code/Software sharing not applicable to this article as no code/software was generated or analysed during the current study.]
